# Defining Drugs in the Electrophysiologist’s World

**DOI:** 10.19102/icrm.2017.080805

**Published:** 2017-08-15

**Authors:** James A. Reiffel


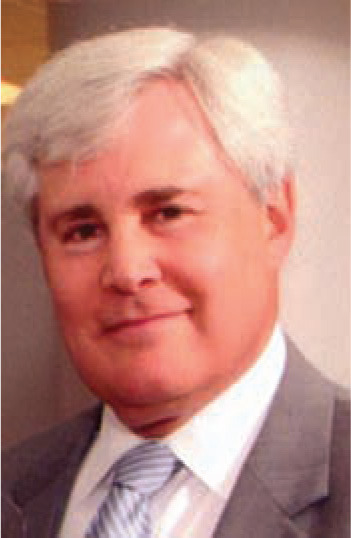


Dear Readers,

With the month of August comes the midpoint between the start of summer and the onset of fall. This year, however, accompanying August is also something else—the appearance of a new section in *The Journal of Innovations in Cardiac Rhythm Management* called *Pharmacological Insights.* This section of the journal will make available to readers manuscripts written on subjects concerning the use of drug therapy in the cardiac electrophysiology patient, including but not limited to anticoagulants and antiarrhythmics. The first of these articles, titled “The Use of Electronic Personal Health Records to Improve Medication Adherence and Patient Engagement: A Randomized Study of Non-Valvular Atrial Fibrillation Patients” by Chen et al., appears in the current issue, and addresses the important subject of medication compliance and possible means to improve it. We all know that medications can only be effective if the patient takes them faithfully as instructed—but do the patients appreciate it and follow our advice? I hope that future manuscripts in the new section of *Pharmacological Insights* will be equally stimulating and informative.

## August and beyond

As time goes on, possible topics in diverse areas that interested authors might consider for inclusion in this new section could include: antiarrhythmic drugs (AAD) under investigation; AAD targets and the specifics of targeting drug therapy to arrhythmia mechanisms; the use of non-antiarrhythmic drugs for arrhythmia therapy; the choice of and roles for agents to reverse anticoagulation; drug-device interactions; drug-drug interactions with AADs and anticoagulants; the use of pharmacodynamics and pharmacokinetics to clinical advantage; the role of genetics in treatment with AADs and anticoagulants; the process from drug development to drug approval; maximizing AAD efficacy while minimizing proarrhythmias; how to understand the United States Food and Drug Administration rules that govern drug marketing; making the selection of AAD in the post-ablation patient; and more. I welcome further suggested topics from the readership. Please feel free to send a possible topic to the editorial office of the *Journal* and/or suggestions for possible authors whom you feel are expert(s) in the proposed arena (including yourself if relevant). Accordingly, we also look forward to manuscripts submitted to us for consideration and will, in addition, seek manuscripts from invited experts.

I trust you will find this new section helpful in your treatment of patients, and stimulating in your personal consideration of approaches to cardiac rhythm management.

Sincerely,

James A. Reiffel, MD, FACC, FAHA, FHRS, FACP

Professor Emeritus of Medicine

Columbia University

New York, NY 10027

